# Physical activity patterns among South-Asian adults: a systematic review

**DOI:** 10.1186/1479-5868-10-116

**Published:** 2013-10-12

**Authors:** Chathuranga D Ranasinghe, Priyanga Ranasinghe, Ranil Jayawardena, Anoop Misra

**Affiliations:** 1 Allied Health Sciences Unit, Faculty of Medicine, University of Colombo, Colombo, Sri Lanka; 2 Department of Pharmacology, Faculty of Medicine, University of Colombo, Colombo, Sri Lanka; 3 Institute of Health and Biomedical Innovation, Queensland University of Technology, Brisbane, Queensland, Australia; 4 Fortis-C-DOC Centre of Excellence for Diabetes, Metabolic Diseases and Endocrinology, Chirag Enclave, New Delhi, India

**Keywords:** Physical activity, Inactivity, South Asia, Adults

## Abstract

Physical activity (PA) has many beneficial physical and mental health effects. Physical inactivity is considered the fourth leading risk factor for global mortality. At present there are no systematic reviews on PA patterns among South Asian adults residing in the region. The present study aims to systematically evaluate studies on PA patterns in South Asian countries. A five-staged comprehensive search of the literature was conducted in Medline, Web of Science and SciVerse Scopus using keywords ‘Exercise’, ‘Walking’, ‘Physical activity’, ‘Inactivity’, ‘Physical Activity Questionnaire’, ‘International Physical Activity Questionnaire’, ‘IPAQ’, ‘Global Physical Activity Questionnaire’ and ‘GPAQ’, combined with individual country names. The search was restricted to English language articles conducted in humans and published before 31st December 2012. To obtain additional data a manual search of the reference lists of articles was performed. Data were also retrieved from the search of relevant web sites and online resources. The total number of hits obtained from the initial search was 1,771. The total number of research articles included in the present review is eleven (India-8, Sri Lanka-2, Pakistan-1). In addition, eleven country reports (Nepal-3, Bangladesh-2, India-2, Sri Lanka-2, Bhutan-1, Maldives-1) of World Health Organization STEPS survey from the South-Asian countries were retrieved online. In the research articles the overall prevalence of inactivity was as follows; India (18.5%-88.4%), Pakistan (60.1%) and Sri Lanka (11.0%-31.8%). STEPS survey reports were available from all countries except Pakistan. Overall in majority of STEPS surveys females were more inactive compared to males. Furthermore, leisure related inactivity was >75% in studies reporting inactivity in this domain and people were more active in transport domain when compared with the other domains. In conclusion, our results show that there is a wide variation in the prevalence of physical inactivity among South-Asian adults within and between countries. Furthermore, physical inactivity in South Asian adults was associated with several socio-demographic characteristics. Majority of South Asian adults were inactive during their leisure time. These Factors need to be considered when planning future interventions and research aimed at improving PA in the region.

## Introduction

South Asia, commonly known as the Indian sub-continent, is home to almost one-fifth of the world’s population and is comprised of many diverse ethnic, linguistic and religious groups [1]. Altogether there are 7 countries in the region namely; Bangladesh, Bhutan, India, Maldives, Nepal, Pakistan and Sri Lanka. Although there are significant cultural differences between regional countries, South Asians are an inherently high-risk group for developing abdominal adiposity, diabetes, cardiovascular diseases [2]. Studies in the United Kingdom have shown that the risk of diabetes is 3 to 5 times higher for immigrants from Bangladesh, India and Pakistan compared with the native white Caucasian population, with an associated increased risk of complications, morbidity and mortality [3]. South Asia has the highest number of patients with diabetes and the prevalence of diabetes among adults is over 10% in many parts of the region [4]. This increased metabolic risk among South Asians appears to be multi-factorial, where unhealthy dietary habits and physical inactivity are coupled with genetic predisposition [5]. Physical inactivity increases the risk of developing abdominal adiposity, diabetes and cardiovascular disease [6]. Furthermore, physical inactivity is considered the fourth leading risk factor for global mortality causing an estimated 3.2 million annual deaths (6% of global deaths) [7].

Physical activity is defined as any bodily movement produced by skeletal muscles that substantially elevates energy expenditure [8]. It is an established fact that physical activity has many beneficial health effects. Regular (≥3 times per week) moderate intensity physical activity such as brisk walking, dancing and gardening decreases the risk of non-communicable disease [9]. Adequate physical activity is also known to increase mental well being [10]. The Harvard alumni study has shown that the alumni mortality rates were significantly lower among those who were physically active, even after adjusting for other lifestyle risk factors [10]. Regular physical activity has been reported to lower blood pressure in adults with hypertension [11]. Physical activity assists in weight loss or a reduction in visceral fat, which could ultimately help in reducing blood pressure [12]. Participation in regular physical activity improves blood glucose control by increasing insulin sensitivity and can prevent or delay onset of type 2 diabetes [13]. Hence it is evident that regular physical activity has numerous cardio-metabolic beneficial effects and the ongoing worldwide epidemic of cardiovascular and metabolic disease is evidence to the fact that the general population is physically inactive.

In the United Kingdom the levels of physical activity have long been known to be lower in the South Asian people than in the general white Caucasian population [14]. The desire to walk, cycle and participate in sports and recreational activity is low among South Asians in comparison with the general population in United Kingdom [14]. Data from the Health Survey for England found that South Asians were 60% less likely than native Caucasians to meet government recommendations for physical activity [15]. Likewise, a systematic review by Fischbacher et al. (2004) examined physical activity levels among South Asians and compared it to the general United Kingdom population and noted that rates were 50–75% less in South Asians [16]. Lack of understanding about benefits, communication gap with health care professionals, cultural beliefs and lack of culturally sensitive facilities are some of the potential barriers for physical activity in South Asians [17]. Swaminathan and Vaz, systematically reviewed the physical activity level of children in India [18]. Although there are several studies from individual South Asian countries on the level of physical activity among adults in each country, at present there are no comprehensive systematic reviews on physical activity patterns among South Asian adults residing in the region. The aim of this exercise is to systematically evaluate published work on physical activity from individual South Asian countries and summarize as for a common region enabling comparison with other regions.

## Methods

A systematic review of published studies reporting physical activity among South Asian adults residing in South Asia was undertaken in accordance with the Preferred Reporting Items for Systematic reviews and Meta-Analyses (PRISMA) statement (Additional file 1) [19].

### Search strategy

A five staged comprehensive search of the literature was conducted in the following databases; PubMed® (U.S. National Library of Medicine, USA), Web of Science® [v.5.4] (Thomson Reuters, USA) and SciVerse Scopus® (Elsevier Properties S.A, USA) for studies published before 31st December 2012. During the first stage the above databases were searched using the following search criteria. The Medline database was searched using the MeSH (Medical Subject Headings) terms ‘Exercise’ and ‘Walking’, with keywords ‘Physical activity’, ‘Inactivity’, ‘Physical Activity Questionnaire’, ‘International Physical Activity Questionnaire’, ‘IPAQ’, ‘Global Physical Activity Questionnaire’ and ‘GPAQ’. The search terms were combined with the names of the individual South-Asian countries; ‘Sri Lanka’, ‘Lanka’, ‘Ceylon’, ‘India’, ‘Bangladesh’, ‘Pakistan’, ‘Nepal’, ‘Bhutan’, ‘Maldives’. The Web of Science database was searched using all of the above search terms in article topic. In the SciVerse Scopus database the above terms were searched in the article title, abstract or keywords.

**Figure 1:**
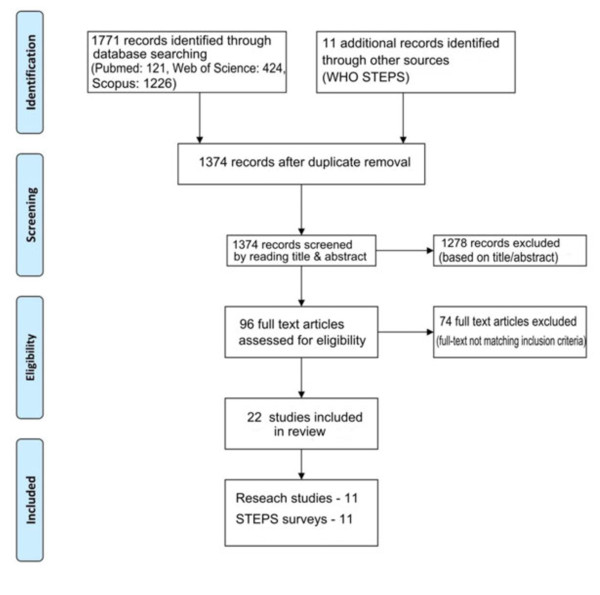
Summarized search strategy.

In the second stage the total hits obtained from searching these three databases were pooled together and duplicates were removed. This was followed by screening of the retrieved articles by reading the article title in the third stage and abstracts in stage four. In the fifth stage individual manuscripts were screened, and those not satisfying inclusion criteria (given below) were excluded. To obtain additional data a manual search of the reference lists of articles selected in stage five was performed. Wherever possible forward citations of the studies retrieved during the literature search was traced and screened for possible inclusion. Furthermore, data were also retrieved from the search of relevant web sites and online resources. This search process was conducted independently by two reviewers (PR and RJ) and the final group of articles to be included in the review was determined after an iterative consensus process. The initial literature search using the above search criteria identified the following number of articles in the respective databases; Medline® (n = 121), Web of Science® (n = 424) and SciVerse Scopus® (n = 1226). The search strategy is summarized in Figure 1.

### Inclusion and exclusion criteria

The following inclusion criteria were used; a) Population-based studies among healthy non-institutionalized human adults (>15 years of age), b) Cross‒sectional study design or being the first phase of a longitudinal study, c) Geographically and temporally defined population from any of the South Asian countries mentioned above, d) Being an original study presenting data on physical activity, e) Evaluating physical activity using questionnaires, f) Published in English, or with detailed summaries in English and g) Peer-reviewed fully published research papers. Studies limited to adults engaged in a particular profession, based in hospitals/institutions and confined to those with diagnosed illnesses were excluded. In addition conference proceedings, editorials, commentaries and book chapters/book reviews were excluded.

### Data extraction

Data were extracted from the included studies by one reviewer using a standardised form and checked for accuracy by a second reviewer. The data extracted from each study were: a) study details (lead author, country/city, year published/year of survey), b) methods (sample size, sampling method, age of subjects in years and type of questionnaire used, definitions), and c) physical activity data (all adults, males, females and domain specific data). Discrepancies in the extracted data were resolved by discussion, with involvement of a third reviewer when necessary.

### Quality assessment

All included research studies were assessed for quality and assigned a rating of either “good” (6–7 points), “fair” (4–5 points) or “poor” (1–3 points) by two reviewers (DCR and RJ). The following seven criteria were used and each one was given a point; 1) appropriateness of research design, 2) appropriate recruitment strategy, 3) adequate response rate (>50%) 4) representativeness of sample, 5) usage of objective and reliable measures to assess physical activity 6) power calculation/justification of numbers and 7) appropriateness of statistical analysis.

## Results

The total number of research studies included in the present review is eleven (India-8, Sri Lanka-2, Pakistan-1). In addition, country reports (n = 11, Nepal-3, Bangladesh-2, India-2, Sri Lanka-2, Bhutan-1, Maldives-1) of World Health Organization (WHO) STEPS survey for non-communicable disease risk factors for the South-Asian countries were retrieved by searching the WHO website (http://www.who.int/chp/steps/reports/en/index.html). The sample size in research studies varied from 416–6,940 and the study setting for research studies were urban (n = 2), rural (n = 3) or both (n = 6). In the STEPS surveys sample sizes were between 2,026 and 44,491. Most research studies used self designed interviewer administered questionnaires for evaluation of physical activity (n = 7). International Physical Activity Questionnaire (IPAQ) was used by 3 research studies and 1 study used WHO STEPS survey tool. All WHO STEPS surveys used Global Physical Activity Questionnaire (GPAQ) for the evaluation of physical activity. Majority of the research studies received a “good” (n = 5) or “fair” (n = 3) ranking on the quality assessment, while only 3 were ranked “poor” (Additional file 2). A separate assessment is not included for the WHO STEPS surveys conducted according to the uniform STEPS study protocol which satisfies all of the above seven criterion used for quality assessment.

### Definition of inactivity

How sedentary lifestyle or inactivity was defined varied significantly in the different studies that used self designed questionnaires to evaluate physical activity (Table 1). Physical Activity Level (PAL) (Estimated total energy expenditure per 24 hrs / basal metabolic rate) was calculated in 3 studies and a PAL for inactivity was defined (<1.5/<1.69) [20-22]. However, there was a wide variation in the definition of physical inactivity in the other studies (Table 1). In the WHO STEPS surveys using the GPAQ, the total of the categorical score in the 3 domains (work, leisure time and transport) classifies an individual into three categories; ‘Inactive’, ‘Moderately active’ and ‘Highly active’. The ‘Inactive’ category includes those who do not perform any physical activity or those reporting some activity, but not enough to meet other categories. The same definition was used in the IPAQ for the ‘Inactive’ category.

**Table 1:** Summary of findings from research studies

**Author [ref] Country Year of study**	**Sample characteristics**	**Assessment tools and definitions**	**Results**
Vaz M et al. [20,21], India, Study year not reported Published 2004 ,2006	Sample size 782 (males 341, females 441), Age 17-70 yrs Urban setting Convenient sampling	Interviewer administered questionnaire, Physical activity level (PAL) = (Daily Total energy expenditure/Estimated basal metabolic rate), Sedentary/Inactive (PAL) <1.5	● Physical activity level (PAL) in adult males 1.22-1.64 and females 1.30-1.56., ● Overall physical activity in oldest group (> 58 yrs) was significantly low and females had low overall physical activity levels than males, ● Discretionary exercise was the highest in the youngest age, ● Women had significantly lower discretionary exercise and higher levels of household chores than males, ● Males and females who did not exercise >20 min/day ranged from 22.6%-60.8% and 40.9%-75.8% across age groups (Lowest in >58 yrs and highest in 36-46 yrs age group), ● People who exercised (recreational) were not active in other domains.
Krishnan A et al. [23], India 2003-2004	Sample size 2828, (Males 1359, Females 1469), Age 15-64 yrs, Rural, Multistage sampling	WHO STEPS like survey	● Inactivity prevalence was 34.2%* (Males - 22.2%, Females -45.5%) and women were more inactive in all domains; ● Inactivity more at leisure time (Males 85.2%, Females 97.3%), and less at transport (Males - 18.8%, Females 45.7%), ● Inactivity at work was Males - 57.2% and Females - 59.9%; ● Physical inactivity was highest in old age (55-64 yrs) and lowest at 35-45 yrs age group
Sugathan TN et al. [24], India, 2003 - 2004	Sample size 6579, (Males 2890, Females 3689), Age 30-74 yrs, Urban and rural, Stratified multistage cluster sampling	Interviewer administered questionnaire, Inactive: Always, carrying out only light/sedentary activities in work + leisure + travel, Inactivity calculation based on a Composite index including work + leisure	● Inactivity prevalence was 22.3% (Males - 22.9%, Females - 21.9% ), ● Urban residents were more inactive (corporation 25.6%, municipality 20.7%) than rural 21.8%, ● Inactivity more at leisure time (74.0%), less at work (31.0%), ● Young (30-34 yrs) were more inactive (24.7%, RR = 1.0) than old (65–74 yrs) (18.9%, RR = 0.7), ● Skilled workers (28.5%, RR = 3.0) and professionals (32.0%, RR = 3.3) more inactive than unskilled (12.3%, RR = 1.0).
Agrawal VK et al. [25], India, Study year not reported, Published 2006	Sample size 416, (Males 218, Females 188), Age >30 yrs, Rural, Random sampling	Interviewer administered questionnaire, Inactive: Doing no or very little activity at work, home or transport and discretionary time	● Inactivity prevalence was 18.5%, ● There was no significant gender difference in prevalence of inactivity, ● In males inactivity was 19.7%, while in females it was 17.0%
Sullivan R et al. [22], India, 2005-2007	Sample size 6,447, (Males 3,768, Females 2,679), Age 17-76 yrs, Urban, rural and migrants, Mixed sampling	Interviewer administered questionnaire, PAL was calculated and categorized, PAL <1.40 extremely inactive, PAL 1.40–1.69 sedentary/lightly active	● Extreme inactivity prevalence 9.7% (Males 7.4%, Females 12.9%), ● Sedentary/lightly active prevalence 62.1% (Males 58.8%, Females 66.7%)
Mittal M et al. [26], India, Year not reported, Published 2011	Sample size 520, (Males 260, Females 260), Age 20-50 yrs, Urban and rural, Random sampling	Interviewer administered questionnaire, Inactive: Sedentary job and no physical exercise or cycling, Moderately inactive: Sedentary job and some but <1 hour physical exercise and/or cycling per week OR Standing job and no physical exercise or cycling	● Prevalence of inactivity 29.4%* (Males 12.7%, Females 46.1%, Urban 29.6%, Rural 29.2%), ● Prevalence of moderate inactivity 21.5%,* (Males 25.7%, Females 17.3%, Urban 30.0%, Rural 13.1%), ● Inactivity was more in Urban and in females, ● Urban females waist circumference reduced (p < 0.05) with increased physical activity, ● BMI showed a steady decline from inactivity to activity
Haldiya KR et al. [27], India, Study year not reported, Published 2010	Sample size 1,825, (Males 650, Females 1175), Age >20 yrs, Rural population	Interviewer administered questionnaire, Sedentary lifestyle: those who had never felt increase heart/respiratory rate after work continued at least for 10 minutes	● 40.0% had a sedentary lifestyle (Males 40.8%, Females 39.7%)
Agrawal R et al. [28], India, 2009-2010	Sample size 544, (Males:Females - 1:1), Age >45 yrs, Urban and rural, Multi stage simple random sampling	Interviewer administered questionnaire, Inactivity: Exercise <30 min/day	● Exercise <30 min/d 88.4% (Urban 88.7%, Rural 88.1%), ● Prevalence of hypertension increased with lack of exercise, ● Prevalence of inactivity 60.1% (Males 52.1%, Females 69.8%)
Khuwaja AK and, Kadir [29], Pakistan, Study year not reported, Published 2010	Sample size 534, (Males 292, Females 242), Age 25–64 yrs, Urban, Systematic random sampling	International Physical Activity Questionnaire	● Females were significantly more inactive than males (OR: 2.1, 95% CI 1.5–3.1, p < 0.001)
Arambepola C et al. [30], Sri Lanka, 2004	Sample size 1,400, (Males 720, Females 680), Age 20–64 yrs, Urban and rural, Multi-stage stratified sampling	International Physical Activity Questionnaire	● Prevalence of inactivity 31.8%* (Males 38.5%, Females 24.7%), ● Inactivity in urban adults 35.2% (Males 41.0%, Females 29.0), ● Inactivity in rural adults (Males 35.0%, Females 19.0%), ● Physical inactivity had a significant association with high BMI among women irrespective of their urban or rural living
Katulanda P et al. [31], Sri Lanka, 2005-2006	Sample size 4,485, (Males 1,772, Females 2,713), Age >18 yrs, Urban and rural, Multi-stage random cluster sampling	International Physical Activity Questionnaire-short version	● Prevalence of inactivity 11.0% (Males 14.6%, Females 8.7%)

* - calculated from available data; *NR* – Not reported; *NA* – Not applicable.

### Findings from research studies

In India the overall prevalence of inactivity was 18.5%-88.4% (Table 1). In Indian males the prevalence of inactivity was 12.7%-66.2%. Majority of the studies (n = 5, 62.5%) reported a prevalence of inactivity < 23% in Indian males. In Indian females the inactivity prevalence was 17.0%-79.6%, while majority of the studies (n = 5, 62.5%) reported it to be > 39.5%. The prevalence of inactivity in urban areas of India was reported as 20.7%-88.7%, while in rural areas it was 6.6%-88.1%. One study conducted in rural India reported that physical inactivity was more at leisure time (males - 85.2%, females - 97.3%), and at work (male - 57.2%, female - 59.9%) and less during transport (male - 18.8%, female - 45.7%) [23]. A similar study in both urban and rural areas of India reported inactivity more at leisure time (74.0%) and less at work (31.0%) [24].

There were two research studies on physical activity from Sri Lanka [30,31]. One study was conducted on a nationally representative sample and the other in the most populous western province of Sri Lanka. Both studies looked at urban and rural settings and Inactivity was defined using a uniformed method (IPAQ). The prevalence of Inactivity was 11% in the national sample and 31.8% in the other study conducted in western province (Table 1). Males (14.6% and 38.5%) were more inactive than females (8.7% and 24.7%). Inactivity of urban adults was 35.2% and higher than rural adults (27.6%) in the study from Western province (Table 1). In the National sample; female gender (OR:2.1), age > 70 (OR:3.8), urban-living (OR:2.5), Muslim ethnicity (Sri Lankan Moor) (OR:2.7), tertiary education (OR:3.6), obesity (OR:1.8), diabetes (OR:1.6), hypertension (OR:1.2) and metabolic syndrome (OR:1.3) all significantly increased odds of being ‘inactive’ [31]. The only study on physical activity conducted in urban Pakistan using IPAQ showed that prevalence of inactivity was 60.1% (males-52.1%, females-69.8%) [29].

### Findings from STEPS surveys

In the STEPS survey reports there was uniformity in the sampling and the definition of inactivity. Overall in majority of studies females were more inactive compared to males (Table 2). Furthermore, leisure related inactivity was >75% in studies reporting inactivity in this domain and people were more active in the transport domain when compared with other domains (Table 2).

**Table 2:** Summary of findings from STEPS surveys

**Country Year of study**	**Sample characteristics**	**Results**							
		**Inactive (%)**					**Inactivity in each domain (%)**		
		**Male**	**Female**	**Urban**	**Rural**	**Total**	**Work**	**Transport**	**Recreation**
Bangladesh 2002	Sample size 11,409	NR	NR	50.1^#^	56.7^#^	52^#^			
	(Male 5,625, Female 5,784)								
	Age 25–64 yrs								
	Urban and rural								
	In capital city - Dhaka								
Bangladesh 2009-2010	Sample size 9,275	10.5	41.3	NR	NR	27.0	45.7	44.5	81.9
	(Male 4,312, Female 4,963)								
	Age > 25 yrs								
	Urban and rural								
Bhutan 2007	Sample size 2,484	49.8	69.6	58.6	NA	58.6	69.0	63.2	78.7
	(Male 1,138, Female 1,346)								
	Age 25–74 yrs								
	Urban								
	In capital city - Thimpu								
India 2003-2005	Sample size 44,491								
	(Male 21,871, Female 22,620)								
	Age 15–64 yrs								
	Urban and rural								
	6 States in India								
	1. Assam	8.3	10.7	26.1*	1.6*	9.5*	28.3*	24.6*	NR
	2. Delhi	25.5	15.5	26.3*	NR	20.4*	62.9*	39.3*	NR
	3. Haryana	16.9	47.6	38.3*	24.6*	32.7*	78.4*	41.9*	NR
	4. Kerala	4.8	4.3	7.6*	5.9*	6.7*	18.4*	31.8*	NR
	5. Maharashtra	7.7	6.1	14.9*	1.3*	6.8*	24.5*	21.1*	NR
	6. Tamil Nadu	16.9	27.2	29.1*	19.9*	22.0*	90.8*	25.1*	NR
India 2007-2008	Sample size 38,064								
	(Male 16,891, Female 21,173)								
	Age 24–64 yrs								
	Urban and rural								
	7 states in India								
	1. Andhra Pradesh	55.9	79.7	77.5	63.8	67.7	NR	NR	NR
	2.Kerala	64.7	86.2	70.8	74.5	75.8	NR	NR	NR
	3. Madhya Pradesh	33.5	52.0	68.3	31.8	42.3	NR	NR	NR
	4. Maharashtra	75.4	87.7	86.1	77.2	81.2	NR	NR	NR
	5. Mizoram	60.9	82.4	79.1	62.5	71.1	NR	NR	NR
	6. Tamil Nadu	57.3	74.2	79.4	61.6	65.8	NR	NR	NR
	7. Uttarakhand	64.6	69.7	91.6	57.6	67.1	NR	NR	NR
Maldives 2004	Sample size 2,026	NR	NR	NR	NA	NR	93.2*	NR	NR
	(Male 934, Female 1,092)								
	Age 25–64 yrs								
	Urban (In Male)								
Nepal 2003	Sample size 2,030	73.6	90.9	NR	NA	82.3*	82.3*	27.6*	94.5*
	(Male 1,010, Female 1,020)								
	Age 25–64 yrs								
	Urban								
Nepal 2004-2005	Sample size 7,792	NR	NR	NR	NR	NR	51.5	19.1	86.0
	(Male 3,674, Female 4,118)								
	Age 15–64 yrs								
	Urban and rural								
Nepal 2007	Sample size 4,328	5.2	5.9	NR	NR	5.5	10.6	19.0	83.2
	(Male 1,907, Female 2,421)								
	Age 15–64 yrs								
	Urban and rural								
	15 of 75 districts								
Sri Lanka 2003	Sample size 3,000	12.1	19.1	NR	NR	15.6	58.3*	NR	94.8*
	(Male 1,500, Female 1,500)								
	Age 15–74 yrs								
	Urban								
	One of nine provinces - Western								
Sri Lanka 2006	Sample size 11,680	17.9	31.9	NR	NR	25.0	NR	NR	NR
	(Male 5,765, Female 5,915)								
	Age 24–64 yrs								
	Urban and rural								
	5 random districts out of all 25 districts								

* - calculated from available data; *NR* – Not reported; *NA* – Not applicable.

Two surveys were done in 2002 (n = 11,409) and 2009 (n = 9,275) in Bangladesh. In 2002, overall prevalence of physical inactivity was 52.3%. However, in this survey physical activity was categorised as light, moderate, heavy based on a likert scale; always, usually, sometimes and never. Inactivity was defined as people who have never done light/moderate activities, which was different to the criteria used in all other WHO STEPS surveys. In the national sample (2009) the overall inactivity prevalence was 27%, with more females (41.3%) being inactive than males (10.5%) (Table 2).

Only one STEPS survey has been carried out in Bhutan in the capital city Thimphu (2007, n = 2,484). The overall inactivity prevalence was 58.6% and females (69.6%) were more inactive than males (49.8%). Two large STEPS surveys were done in India in 2003–2005 (6 states, n = 44,491) and 2007–2008 (7 states, n = 39,064). In 2003 the overall prevalence of physical inactivity was 15.8% and in 2007 it was 72.3%. This variation could be attributed to different states used in the two surveys. However Kerala and Maharashtra states were evaluated in both surveys, and the overall prevalence of physical inactivity in Kerala/Maharashtra has increased from 6.7%/6.8% in 2003 to 75.8%/81.2% in 2007. Male and Female physical inactivity in 2003 was 12.6% and 18.9% respectively. In 2007 prevalence of Male and Female physical inactivity was 58.5% and 75.7% respectively. Overall females were more inactive than males and urban residents were more inactive than rural (Table 2). The only STEPS survey conducted in Maldives (2004) has been conducted in its’ capital city Malē (n = 2026). More than 90% (Males 91.2%, Females 94.6%) of the respondents reported that they are physically inactive at work.

Three STEPS surveys have been conducted in Nepal. In 2003 only an urban sample was taken from the capital city (n = 2,030). In later STEPS surveys both urban and rural samples were studied (2004 n = 7,792 and 2007 n = 4,328). The 2003 survey reported an overall inactivity of 82.9% (Males 74.6%, Females 91.2%) in the urban residents. However in contrast the 2007 national sample (conducted in 15 out of 75 districts) reported that overall inactivity was only 5.5% (Males 5.2%, Females 5.9%) (Table 2). In Sri Lanka two STEPS surveys were done in 2003 (single province, n = 3,000) and 2006 (five districts, n = 11,680). In 2003 overall prevalence of physical inactivity was 15.6% and in 2006 it has increased to 25%. Females (2003–19.1%, 2006–31.9%) were more inactive than males (2003–12.1%, 2006–17.9%) in both studies.

## Discussion

To our knowledge this is the first systematic review evaluating physical inactivity and physical activity patterns among adults from South Asian countries. Published research articles evaluating physical activity prevalence were only available from India, Sri Lanka and Pakistan. In contrast STEPS survey data on physical activity were available from all countries except Pakistan. All research studies from the region collectively evaluated physical activity of 26,360 adults, while STEPS surveys contained data on 136,579 adults. We observed a marked variation between research studies in the definition of physical inactivity and in the questionnaires used. Hence, comparisons between studies were undertaken with caution. This barrier was overcome in the STEPS surveys as they used the GPAQ to evaluate physical activity. It is important that future researchers try and adhere to this uniformity in order to derive intra- and inter-regional comparable data and observe secular trends in physical activity. The present study mainly focuses on the prevalence of physical inactivity and its variations with gender and area of residence. We also tried to identify inactivity levels in different domains (work, transport, leisure) as occupations, transport methods, social and cultural values differ in this region compared to other regions and the developed world.

We observed several socio-economical factors associated with physical inactivity. Skilled workers and professionals were more inactive than unskilled workers in the region [24]. Similarly, higher education was a significant factor associated with physical inactivity [31]. Among South Indians, Vaz et al. reported that people engaging in recreational exercise were inactive in other domains [20,21]. Gender is also an important factor to determining physical activity levels of a population. Several studies reported higher physical inactivity among South Asian women [23,25,29,31,32]. Cultural expectations may restrict the participation of women in certain forms of physical activity in some religious and ethnic groups in the region. The traditional role of South Asian women in taking care of household work and supporting extended family members may limit the time available for them to engage in physical activity, in particularly leisure time physical activities. Low physical activity is one of the contributing risk factors for the higher obesity levels seen among Asian Indians [33]. A qualitative study conducted among South Asians living in United Kingdom found that understanding external motivators and social context of their lives is very crucial for developing successful physical activity interventions [34]. Hence, although exercise is promoted in public health campaigns to increase the overall physical activity level of a population, it is important to understand socio-economical factors associated with different populations in order to deliver effective physical activity interventions.

The WHO STEPwise approach was introduced for chronic disease risk factor surveillance in order to close the gap in the availability of data from developing countries and to strengthen their capacity to conduct such surveillances [35]. The GPAQ used in these surveys not only gave uniformity to the gathered data, it also allowed meaningful comparisons. The GPAQ is known to be reliable for surveys in developing countries, adaptable to incorporate cultural differences [36]. The initial STEPS surveys from the region were mostly in sub-populations and confined to logistically favourable areas like capitals. Sri Lanka (2006), Nepal (2007) and Bangladesh (2009) (Inactivity: 25%, 5.5% and 27% respectively) had nationally representative samples in subsequent surveys. Prevalence of overall inactivity reduced in these subsequent surveys as the sample dispersed from populations in urban capitals to the rural parts of the countries. This highlights the effect of urbanity on physical activity. However, in the Sri Lankan STEPS surveys inactivity prevalence has increased in the second survey (2006), which used a national sample. This is probably due to the initial survey (2003) being conducted in more rural areas of the Western province of Sri Lanka, in contrast to Nepal and Bangladesh where the initial surveys were from more urbanised capitals.

The two large STPES surveys (2003, 2007) from India contrasted in the prevalence of inactivity, possibly attributable to subjective variations in data collection, inter-state variations and differences in time and season of data collection. However, data from Kerala and Maharashtra states which were studied in both surveys also showed marked variation. This could be due to differences in the sampling frames used. However such dramatic increases in inactivity over a short period warrants further investigation. The only surveys conducted in urban capitals of Maldives and Bhutan had significantly higher prevalence of inactivity. In Bhutan inactivity was lower compared to Maldives due to it being mostly an agrarian society with nearly 50% of the labour force engaged in agriculture as compared to Maldives where most are involved in the tourism industry [37]. The most active regional country in the STEPS surveys was Nepal, possibly due to 80% of the Nepalese population being involved in agriculture [38]. In all regional countries work and transport related activity was higher compared to leisure time activity. Hence it is important to consider individual country profiles, proportions of urbanity and cultures when delivering public health messages focused on leisure time activity.

Comparable nationally representative country data are available from the WHO World Health Survey (IPAQ questionnaire) conducted in 2002–2003, where the male and female inactivity prevalence was as follows; India 9.3% & 15.2%, Nepal 6.7% & 9.7% and Sri Lanka 7.3% & 13.8% [39]. The data are fairly comparable to those from WHO STEPS surveys in India (2003) and Nepal (2007). In contrast in Sri Lanka inactivity has markedly increased from the 2002 World Health Survey to the 2006 STEPS survey. Several studies show that IPAQ overestimates inactivity prevalence and under estimates prevalence in rural areas of developing countries [32,40,41]. The STEPS surveys have also been conducted in 80 developing countries around the world allowing for a meaningful comparison across regions [35]. In the Western Pacific region (19 countries) inactivity prevalence ranged from 7.5% in Mongolia to 75.3% in Cook Islands with majority of countries falling between 40%-66%. Eastern Mediterranean (14 countries) prevalence of inactivity ranged from 30%-60%. In Africa (31 countries) lowest prevalence of inactivity was observed in Mozambique (6.4%) and highest in Zimbabwe (79.3%). In nearly half of the African countries inactivity was <29%. East Asian countries like Myanmar (12.7%) and Indonesia (22.6%) had prevalence’s of inactivity comparable with the South Asia and Africans. With the available data we observe that the persons residing in South Asian region are more active compared with the Western Pacific and Mediterranean countries, lying in parallel with East Asia and Africa. In developing countries with agrarian economies inactivity was low irrespective of the region (Nepal, Mongolia, Malawi, Mozambique and Cambodia). However the inactivity prevalence seems to be on the rise in most Western Pacific countries due to recent economic transitions. Hence, countries in the South Asian region in economic transition also need public health planning to enable the people to maintain their already existing active life styles. We also observe that females were more inactive in the South Asian region when compared to males, a finding which is seen in most other regions and even in developed countries.

The International Prevalence Study conducted in 2002–2004 using IPAQ categorized inactivity to be least in China (6.9%), New Zealand (12.2%), Canada (13.7%), USA (15.9%) and Australia (17.2%) [42]. We observe that South Asian activity levels were in parallel or sometimes better than these developed countries which have longer physical activity promotion strategies, mainly targeted at improving leisure time physical activity. However with increased urbanisation and busy work schedules the leisure time is also reduced. In the South Asian region we observed that activity during work and transport is higher than leisure time activity. Promoting leisure time activity has also become a challenge in South Asia due to cultural and attitudinal barriers. Hence public health messages in the region should be directed at improving all domains of activity with work and transport policies of the countries supporting them.

One of the strengths of the current systematic review is the comprehensive and easily replicable search strategy applied to 3 major medical databases. In addition we systematically selected the studies through the application of well-defined inclusion/exclusion criteria. We would also like to highlight several limitations in the present review. Firstly, most of studies were limited to regional populations. As South Asian countries have considerably diverse ethnic groups, the regional findings may not be generalized to whole country or for the entire South Asian region. Secondly, there is no uniformity on physical activity assessment tools. Although several of studies have used IPAQ, differences in the formats used limit comparability. For instance, Arambepola et al. has used longer version of IPAQ whereas Katulanda et al. has used a short version [30,31]. Thirdly, there is clear heterogeneity on the definition of physical inactivity/sedentary life style and physical activity levels. Furthermore, only limited number of articles reported data on the sub-domains of physical activity (work, transport and leisure). In addition, although many studies have shown that resistance exercise improves overall health and that it is beneficial for South Asians with Diabetes at present there is only very limited data available for South Asians [43,44].

## Conclusions

There is a wide variation in the prevalence of physical inactivity among South-Asian adults within and between countries. Hence it is difficult to comment about the overall prevalence of physical inactivity in the region. In the South Asian regions females, skilled workers, professionals and those with higher education were more inactive. Majority of South Asian adults were inactive during their leisure time. These factors need to be considered when planning future interventions and research aimed at improving PA in the region. However, future researchers should try and adhere to uniformity in definitions and assessment tools in order to derive intra- and inter-regional comparable data and observe secular trends.

## Abbreviations

GPAQ: Global physical activity questionnaire; IPAQ: International physical activity questionnaire; MeSH: Medical subject heading; MET: Metabolic equivalents; OR: Odds ratio; PAL: Physical activity level; PRISMA: Preferred reporting in systematic reviews & Meta-Analysis; WHO: World Health Organization.

## Competing interests

The authors declare that they have no competing interests.

## Authors’ contributions

DC, PR and RJ made substantial contribution to conception and study design. DC, PR and RJ were involved in data collection. DC, PR, RJ and AM were involved in refining the study design, statistical analysis and drafting the manuscript. DC, PR, RJ and AM critically revised the manuscript. All authors read and approved the final manuscript.

## Supplementary Material

Additional file 1 PRISMA 2009 checklist. Click here for file

Additional file 2 Assessment of Quality of the included Research Studies. Click here for file

## References

[B1] Gupta M Singh N Verma S South Asians and cardiovascular risk: What clinicians should know Circulation 2006 113 25 e924 e929 10.1161/CIRCULATIONAHA.105.583815 16801466

[B2] Eapen D Kalra GL Merchant N Arora A Khan BV Metabolic syndrome and cardiovascular disease in South Asians Vasc Health Risk Manag 2009 5 731 743 19756165 10.2147/vhrm.s5172 PMC2742703

[B3] Erens B Primatesta P Prior G The health of minority ethnic groups ′99 methodology & documentation 2001 London: The health survey for england

[B4] Jayawardena R Ranasinghe P Byrne NM Soares MJ Katulanda P Hills AP Prevalence and trends of the diabetes epidemic in South Asia: a systematic review and meta-analysis BMC Public Health 2012 12 380 10.1186/1471-2458-12-380 22630043 PMC3447674

[B5] Gupta M Brister S Is South Asian ethnicity an independent cardiovascular risk factor? Can J Cardiol 2006 22 3 193 197 10.1016/S0828-282X(06)70895-9 16520847 PMC2528919

[B6] Qin L Corpeleijn E Jiang C Thomas GN Schooling CM Zhang W Cheng KK Leung GM Stolk RP Lam TH Physical activity, adiposity, and diabetes risk in middle-aged and older Chinese population: The Guangzhou Biobank Cohort Study Diab Care 2010 33 11 2342 2348 10.2337/dc10-0369 PMC2963492 20713687

[B7] World Health Organization Global health risks: Mortality and burden of disease attributable to selected major risks 2009 Geneva: World Health Organization

[B8] Aust NZJ MedCaspersen CJ Powell KE Christenson GM Physical activity, exercise, and physical fitness: Definitions and distinctions for health-related research Public Health Rep 1985 100 2 126 131 3920711 PMC1424733

[B9] Willett WC Koplan JP Nugent R Dusenbury C Puska P Gaziano TA Jamison DT, Breman JG, Measham AR, Alleyne G, Claeson M, Evans DB, Jha P, Mills A Prevention of chronic disease by means of diet and lifestyle changes Disease control priorities in developing countries 2006 2 Washington (DC): Musgrove P

[B10] Fox KR The influence of physical activity on mental well-being Public Health Nutr 1999 2 3A 411 418 10610081 10.1017/s1368980099000567

[B11] Ishikawa K Ohta T Zhang J Hashimoto S Tanaka H Influence of age and gender on exercise training-induced blood pressure reduction in systemic hypertension Am J Cardiol 1999 84 2 192 196 10.1016/S0002-9149(99)00233-7 10426339

[B12] Reaven PD Barrett-Connor E Edelstein S Relation between leisure-time physical activity and blood pressure in older women Circulation 1991 83 2 559 565 10.1161/01.CIR.83.2.559 1991374

[B13] Hamman RF Wing RR Edelstein SL Lachin JM Bray GA Delahanty L Hoskin M Kriska AM Mayer-Davis EJ Pi-Sunyer X Effect of weight loss with lifestyle intervention on risk of diabetes Diabetes Care 2006 29 9 2102 2107 10.2337/dc06-0560 16936160 PMC1762038

[B14] Zaman MJ Jemni M South Asians, physical exercise and heart disease Heart 2011 97 8 607 609 10.1136/hrt.2010.214908 21156678

[B15] Williams ED Stamatakis E Chandola T Hamer M Assessment of physical activity levels in South Asians in the UK: Findings from the Health Survey for England J Epidemiol Community Health 2011 65 6 517 521 10.1136/jech.2009.102509 20525752

[B16] Fischbacher CM Hunt S Alexander L How physically active are South Asians in the United Kingdom? A literature review J Public Health (Oxf) 2004 26 3 250 258 10.1093/pubmed/fdh158 15454592

[B17] Horne M Tierney S What are the barriers and facilitators to exercise and physical activity uptake and adherence among South Asian older adults: A systematic review of qualitative studies Prev Med 2012 55 4 276 284 10.1016/j.ypmed.2012.07.016 22846506

[B18] Swaminathan S Vaz M Childhood physical activity, sports and exercise and noncommunicable disease: A special focus on India Indian J Pediatr 2013 80 1 63 70 10.1007/s12098-011-0590-y 22791355

[B19] Moher D Liberati A Tetzlaff J Altman DG Group P Preferred reporting items for systematic reviews and meta-analyses: The PRISMA statement BMJ 2009 339 b2535 10.1136/bmj.b2535 19622551 PMC2714657

[B20] Vaz M Bharathi AV Kurpad AV Exercising’ but not active: Implications for physical activity counselling Natl Med J India 2006 19 6 345 17343023

[B21] Vaz M Bharathi AV Perceptions of the intensity of specific physical activities in Bangalore, South India: Implications for exercise prescription J Assoc Physicians India 2004 52 541 544 15645977

[B22] Sullivan R Kinra S Ekelund U Bharathi AV Vaz M Kurpad A Collier T Reddy KS Prabhakaran D Ben-Shlomo Y Socio-demographic patterning of physical activity across migrant groups in India: results from the Indian Migration Study PLoS One 2011 6 10 e24898 10.1371/journal.pone.0024898 22022366 PMC3194815

[B23] Krishnan A Shah B Lal V Shukla DK Paul E Kapoor SK Prevalence of risk factors for non-communicable disease in a rural area of Faridabad district of Haryana Indian J Public Health 2008 52 3 117 124 19189832

[B24] Sugathan TN Soman CR Sankaranarayanan K Behavioural risk factors for non communicable diseases among adults in Kerala, India Indian J Med Res 2008 127 6 555 563 18765874

[B25] Agrawal VK Basannar DR Sing RP Dutt M Abraham D Mustafa MS Coronary risk factors in a rural community Indian J Public Health 2006 50 1 19 23 17193754

[B26] Mittal M Arora M Bachhel R Kaur N Sidhu RS Physical activity, indices of obesity and mean arterial blood pressure: Does place of living matters? rural vs urban J Clin Diagn Res 2011 5 5 1038 1042

[B27] Haldiya KR Mathur ML Sachdev R Lifestyle-related risk factors for cardiovascular disease in a desert population of India Curr Sci 2010 99 2 190 195

[B28] Agrawal R Chaturvedi M Singh S Gupta SC An epidemiological study of dietary and exercise habits as correlates of hypertension in persons aged 45 years and above in Agra District Indian J Comm Health 2012 24 2 91 96

[B29] Khuwaja AK Kadir MM Gender differences and clustering pattern of behavioural risk factors for chronic non-communicable diseases: Community-based study from a developing country Chronic Illn 2010 6 3 163 170 10.1177/1742395309352255 20444764

[B30] Arambepola C Allender S Ekanayake R Fernando D Urban living and obesity: Is it independent of its population and lifestyle characteristics? Trop Med Int Health 2008 13 4 448 457 10.1111/j.1365-3156.2008.02021.x 18331534

[B31] Katulanda P Jayawardana R Ranasinghe P Rezvi Sheriff M Matthews DR Physical activity patterns and correlates among adults from a developing country: The Sri Lanka Diabetes and Cardiovascular Study Public Health Nutr 2013 16 9 1684 1692 10.1017/S1368980012003990 22995708 PMC10271739

[B32] Ekelund U Sepp H Barge S Criterion-related validity of the last 7-day, short form of the International Physical Activity questionnaire in Swedish adults Public Health Nutr 2006 9 258 265 16571181 10.1079/phn2005840

[B33] Chopra SM Misra A Gulati S Gupta R Overweight, obesity and related non-communicable diseases in Asian Indian girls and women Eur J Clin Nutr 2013 67 7 688 696 10.1038/ejcn.2013.70 23612512

[B34] Jepson R Harris FM Bowes A Robertson R Avan G Sheikh A Physical activity in South Asians: An in-depth qualitative study to explore motivations and facilitators PLoS One 2012 7 10 e45333 10.1371/journal.pone.0045333 23071511 PMC3468573

[B35] World Health Organization WHO Chronic diseases and health promotion, STEPS country reportshttp://www.who.int/chp/steps/reports/en/index.html

[B36] Armstrong TA Bull FC Development of the Global Physical Activity Questionnaire (GPAQ) J Public Health 2006 14 66 70 10.1007/s10389-006-0024-x

[B37] Central Intelligence Agency The world factbook 2013 https://www.cia.gov/library/publications/the-world-factbook/index.html

[B38] Ertur O The need for a national urbanization policy in Nepal Asia Pac Popul J 1994 9 3 19 36 12319088

[B39] Guthold R Ono T Strong KL Chatterji S Morabia A Worldwide variability in physical inactivity a 51-country survey Am J Prev Med 2008 34 6 486 494 10.1016/j.amepre.2008.02.013 18471584

[B40] Ainsworth BE Macera CA Jones DA Comparison of the 2001 BRFSS and the IPAQ physical activity questionnaires Med Sci Sports Exerc 2006 38 1584 1592 10.1249/01.mss.0000229457.73333.9a 16960519

[B41] Craig CL Marshall AL Sjöström M International physical activity questionnaire: 12-country reliability and validity Med Sci Sports Exerc 2003 35 1381 1395 10.1249/01.MSS.0000078924.61453.FB 12900694

[B42] Bauman A Bull F Chey T Craig CL Ainsworth BE Sallis JF Bowles HR Hagstromer M Sjostrom M Pratt M The international prevalence study on physical activity: results from 20 countries Int J Behav Nutr Phys Act 2009 6 21 10.1186/1479-5868-6-21 19335883 PMC2674408

[B43] Hills AP Shultz SP Soares MJ Byrne NM Hunter GR King NA Misra A Resistance training for obese, type 2 diabetic adults: a review of the evidence Obes Rev 2010 11 10 740 749 20003071 10.1111/j.1467-789X.2009.00692.x

[B44] Misra A Alappan NK Vikram NK Goel K Gupta N Mittal K Bhatt S Luthra K Effect of supervised progressive resistance-exercise training protocol on insulin sensitivity, glycemia, lipids, and body composition in Asian Indians with type 2 diabetes Diabetes Care 2008 31 7 1282 1287 10.2337/dc07-2316 18316394 PMC2453659

