# Supplementary material for: Physical activity patterns among South-Asian adults: a systematic review
Source: Int J Behav Nutr Phys Act. 2013 Oct 12;10:116. doi: 10.1186/1479-5868-10-116 (PMC3854453; doi:10.1186/1479-5868-10-116)
Supplement: Additional file 2 — Assessment of Quality of the included Research Studies. [file 1479-5868-10-116-S2.doc]

**Additional File 2: Assessment of Quality of the included Research Studies**

| Author | Appropriate Research Design? | Appropriate Recruitment Strategy? | Response  Rate? | Is Sample  Representative? | Objective  & Reliable  Measures | Power  Calculation/  Justification  of Numbers | Appropriate  Statistical  Analysis? | Quality  Indicator  Score |
| --- | --- | --- | --- | --- | --- | --- | --- | --- |
| Vaz M et al.  India | No | No | NR | Unclear | No | NR | Yes | 1/7 |
| Krishnan A et al.  India | Yes | Yes | NR | Yes | Yes | NR | Yes | 5/7 |
| Sugathan TN et al.  India | Yes | Yes | 95.1% | Yes | No | Yes | Yes | 6/7 |
| Agrawal VK et al.  India | Yes | Yes | 97.5%* | Yes | No | Yes | Yes | 6/7 |
| Sullivan R et al.  India | No | No | 85%* | Unclear | Yes | NR | Yes | 3/7 |
| Mittal M et al.  India | No | Yes | NR | Unclear | No | NR | Yes | 2/7 |
| Haldiya KR et al.  India | Yes | Yes | 62.7% | Unclear | No | NR | Yes | 4/7 |
| Agrawal R et al.  India | Yes | Yes | 98.3%* | Yes | No | Yes | Yes | 6/7 |
| Khuwaja AK and  Kadir  Pakistan | Yes | No | 88.4%* | Unclear | Yes | NR | Yes | 4/7 |
| Arambepola C et al.  Sri Lanka | Yes | Yes | 96.3% | Yes | Yes | NR | Yes | 6/7 |
| Katulanda P et al.  Sri Lanka | Yes | Yes | 91.1% | Yes | Yes | NR | Yes | 6/7 |

NR – Not Reported, * - Calculated from available data
